# Assessing the potential of *Chlorella* sp. phycoremediation liquid digestates from brewery wastes mixture integrated with bioproduct production

**DOI:** 10.3389/fbioe.2023.1199472

**Published:** 2023-06-14

**Authors:** Sen Wang, Qiang Zhao, Haiyang Yu, Xinxin Du, Ting Zhang, Tongxin Sun, Wenlu Song

**Affiliations:** New Energy Research Institute, Jining University, Qufu, Shandong, China

**Keywords:** digestate, *Chlorella* sp., brewer’s grains, biomass composition, photosynthetic fluorescence parameters, brewery wastewater

## Abstract

Digestates from different anaerobic digesters are promising substrates for microalgal culture, leading to effective wastewater treatment and the production of microalgal biomass. However, further detailed research is needed before they can be used on a large scale. The aims of this study were to investigate the culture of *Chlorella* sp. in Digestate_M_ from anaerobic fermentation of brewer’s grains and brewery wastewater (BWW) and to explore the potential use of the biomass produced under different experimental conditions, including diverse cultivation modes and dilution ratios. Cultivation in Digestate_M_ initiated from 10% (v/v) loading, with 20% BWW, obtained maximum biomass production, reaching 1.36 g L^−1^ that was 0.27g L^−1^ higher than 1.09 g L^−1^ of BG11. In terms of Digestate_M_ remediation, the maximum removal of ammonia nitrogen (NH_4_^+^-N), chemical oxygen demand, total nitrogen, and total phosphorus reached 98.20%, 89.98%, 86.98%, and 71.86%, respectively. The maximum lipid, carbohydrate, and protein contents were 41.60%, 32.44%, and 27.72%, respectively. The growth of *Chlorella* sp. may be inhibited when the Y(II)–F_v_/F_m_ ratio is less than 0.4.

## 1 Introduction

In recent years, the fast development of human society has led to the rapid consumption of various nonrenewable resources and people’s demand for sustainable new sources of food and energy. The versatility of microalgal biomass is exactly what people need now given its ability to assimilate carbon dioxide through photosynthesis and high areal productivity on nonarable land (Cheng et al., 2016). The cultivation of microalgae is often coupled with the treatment of wastewater to obtain microalgal biomass and to treat the wastewater (Zhang et al., 2016), which can solve the problem of water shortage and environmental pollution (Shahid et al., 2019). The direct discharge of anaerobic digestive effluents, owing to their high nutrient value, not only causes environmental pollution but also wastes various nutrient elements in the digestive effluents; in recent years, extensive investigations have been conducted to find the digestive effluents suitable for the growth of microalgae (Koutra et al., 2018a; Alavijeh et al., 2020). It is a good strategy to treat digested effluents with microalgae (Chuka-ogwude et al., 2020). The high pH value, high turbidity and low C/N ratio of effluents will affect the growth of microalgae, and the inhibiting factors will also vary with the anaerobic fermentation substrate, which brings challenges to the large-scale treatment digestives by microalgae (Bonetta et al., 2014; Xia and Murphy, 2016). To solve this difficulty, researchers often carry out pretreatment of digested effluents, such as physical adsorption, flocculation, biological contact oxidation, and ozone treatment (Kim et al., 2014). Although these methods can reduce inhibitors for the survival of microalgae in digested effluents, the nutrients in effluents are greatly reduced, and the cost is high (Qian et al., 2019). Dilution of digested effluents is also a pretreatment method. Different dilution ratios, effluent types, and experimental conditions will change the production and biochemical components of microalgal biomass (Mayers et al., 2017; Romero-Villegas et al., 2018; Zhao et al., 2019). Therefore, applied conditions and the biological composition of microalgae obtained from different digested effluents play critical roles in subsequent applications (Eppink et al., 2017).

*Chlorella* sp. comprises various nutritional modes: autotrophy, photoheterotrophy, and heterotrophy (Zhu, 2015). This property makes *Chlorella* have the potential to utilize organic wastewater and adapt to harsh environments (Chew et al., 2018). Moreover, *Chlorella* biomass is rich in polysaccharides, proteins, lipids, and other organic matter, which have broad application prospects in food, energy, materials, and other fields (Safi et al., 2014; Sakarika and Kornaros, 2019), and it is one of the few microalgae that can be cultivated on a large scale (Stiles et al., 2018). About different pretreatment methods of digestate, Franco et al. (Tfab et al., 2020) used a *Chlorella*–bacteria combined system to treat waste food biogas slurry. When the concentration of effluents reached 100%, the removal rates for total organic carbon, total nitrogen (TN), and total phosphorus (TP) were still as high as 85.00% ± 3%, 73.00% ± 3%, and 28.00% ± 16%, respectively. Through the pretreatment of the digestate effluent with indigenous bacteria in the anaerobic digestion liquid of pig manure, the modification of waste liquid by *Chlorella vulgaris* was also enhanced, and the biomass yield of *C. vulgaris* was increased (Gu et al., 2021). Concerning the use of digestate, Dinnebier et al. (2021) used *Chlorella sorokiniana* to treat swine digestate, reaching the maximum biomass productivity of 198 mg L^−1^ d^−1^ (dry weight) and removal of 90.00% and 70.00% of TP and TN, respectively. The protein content of biomass was as high as 59.50%. With a three-step process for treating anaerobically digested swine manure, the maximum biomass concentration of *C. vulgaris* reached 2.33 g L^−1^, accompanied by the removal of 97.20%, 94.00%, 99.70%, and 99.90% of chemical oxygen demand (COD), TN, TP, and ammonia nitrogen (NH_4_^+^-N) (Wang et al., 2019). Recently, an integrated effective treatment process for digestate from agro-industrial and municipal organic wastes, combining different dilution ratios, different culture models, and nutrient supplement of digestate, resulted in the maximum biomass concentration of *C. vulgaris* up to 1.63 g L^−1^ and organic carbon, TN, and TP removal rates of 92.00%, 77.00%, and 94.00%, respectively (Koutra et al., 2021). Through the addition of 2 g L^−1^ of a glycerol solution to digested municipal wastewater as a supplementary carbon source, the growth rate and lipid production rate of *C. vulgaris* under mixotrophic conditions reached 0.064 g L^−1^ d^−1^ and 20.4 mg L^−1^ d^−1^, respectively (Ge et al., 2018). About the use of wastewater, using *Chlorella sorokiniana CY-1* to treat waste molasses showed a reduction in COD, total suspended solid, and nitrate content of 61.48%, 63.94%, and 63.79%, respectively and 155.0 mg L^−1^ of molasses concentration medium obtained the highest biomass productivity of 160.9 mg L^−1^ d^−1^ (Guo et al., 2020). Moreover, *Chlorella* sp. and *Chlorococcum* sp. were effectively cultivated in industrial wastewater with varying concentrations of coal-fired flue gas from 1% to 10% CO_2_, resulting in the highest biomass production of 1.52 g L^−1^ and COD, ammonia nitrogen (NH_4_^+^-N), TP, and nitrate maximum removal rates of 91.9%, 95.9%, 98.8%, and 100%, respectively (Yadav et al., 2019). Comparing the effects of urban wastewater (UWW) and Bold’s basal medium (BBM) on microalgal growth, the lipid productivity (LP) in UWW was 1.15 fold that in BBM, and the largest fat yield in UWW was 14.31 mg L^−1^ d^−1^, which simultaneously had removal rates of up to 87.90% and 98.40% for TN and TP by *C. vulgaris*, respectively (Singh et al., 2017). Microalgae can grow under different culture modes, such as photoautotrophic cultivation widely used in microalgal cultivation, heterotrophic cultivation that mainly uses organic matter to grow (Chojnacka and Marquez-Rocha, 2004), and mixotrophic cultivation using organic matter and inorganic matter at the same time (Wang et al., 2014). Cultivation modes will affect the metabolism pathway and biomass composition of *C. vulgaris* (Changling et al., 2014).

China is a major producer of beer, with output reaching 35.62 million tons in 2021 despite the impact of the COVID-19. Each ton of beer produced will generate 3–10 tons of brewery wastewater (BWW) (Fillaudeau et al., 2006) and 0.25 tons of brewer’s grains (BWG) (Aliyu and Bala, 2011). There are different treatment and utilization methods for BWW and BWG. Lu et al. (2019) obtained photosynthetic bacteria biomass while treating BWW, and the maximum yield of the biomass could reach 483.5 mg L^−1^ d^−1^, realizing 420.9, 177.6, and 2.53 mg g^−1^ of protein, polysaccharide, and carotenoid in the photosynthetic bacteria biomass, respectively. Exploring the cultivation of *Chlorella* sp. in BWW, Wang et al. (2022) obtained not only the maximum biomass concentration of 2.03 g L^−1^ but also the maximum lipid, carbohydrate, and protein contents of 36.19%, 36.51%, and 19.77%, respectively, with the maximum removal of NH_4_^+^-N, COD, TN, and TP reaching 94.38%, 88.52%, 96.71%, and 98.68%, respectively. Previous studies have reported the production of methane (Chen et al., 2016) and hydrogen (Shi et al., 2010) by anaerobic fermentation of BWW, but the production of biogas by anaerobic fermentation of BWW–BWG mixtures has not been studied. The main objective of this study was to cultivate *Chlorella* sp. in effluents from anaerobic digestion of BWW–BWG mixtures and under various culture conditions. The potential of microalgal treatment of digestate was investigated, and the potential uses of microalgal biomass were explored. The application treatment methods included different culture models (mixotrophic and heterotrophic conditions) and dilution ratios of digestate. In the experimental process, microalgal biomass production and BWW–BWG mixture digestate (Digestate_M_) remediation were evaluated, accompanied by the investigation of photosynthetic fluorescence parameters and microalgal biomass components, including pigments, proteins, carbohydrates, and lipids.

## 2 Materials and methods

### 2.1 Digestate_M_ collection and treatment

The digested effluents were produced by the process of single-phase anaerobic fermentation, which was carried out at medium temperature (42°C) on lab-scale. Mixtures of BWW and BWG were treated at HRT of 45 days.

After the above experiment, the fermentation mixture was filtered using a 100-mesh filter bag, and the digested effluents were collected and centrifuged (XL5A, XIANG LI) for 10 min at 4,000 rpm. A sand core filter, combined with a 0.45 μm filter membrane, was also used to filter the supernatant to remove the solid components.

Subsequently, the main physicochemical characteristics of Digestate_M_ were analyzed, as detailed in Table 1. Digestate_M_ was kept in a refrigerator at −18°C.

**TABLE 1:** Physicochemical properties of BWW–BWG mixture digestate (Digestate_M_) and brewery wastewater (BWW). Data are means ± SD (*n* = 2).

Mean value or concentration (mg L^−1^)	Digestate_M_	BWW
OD_550_	2.37 ± 0.00	0.33 ± 0.01
pH	9.03 ± 0.05	7.83 ± 0.06
TS	10381 ± 0.46	480.0 ± 0.03
VS	3862 ± 0.35	264.0 ± 0.10
COD	45720.0 ± 0.40	995.23 ± 0.15
TN	2050.8 ± 0.72	63.62 ± 0.03
TP	162.93 ± 0.45	1.15 ± 0.02
NH_4_^+^-N	1450.17 ± 0.86	44.33 ± 0.21

### 2.2 *Chlorella* sp. cultures and experimental conditions

*Chlorella* sp. was obtained from the Freshwater Algae Culture Collection at the Institute of Hydrobiology, Wuhan, China. First, the obtained *Chlorella* sp. was incubated in BG11 AGAR medium at 25°C for 7 days in a sterile procedure (SW-CJ-1F ISO-5, AIRTECH) to obtain single algal strains. A single algal strain was selected and placed in 200 mL triangular bottles with 50 mL of BG11 medium for static culture, maintaining the extended culture for 7 days. After the expanded culture, *Chlorella* sp. was cultured by passage, and the fourth generation was used as experimental inoculum for subsequent experiments. The experiment was conducted in an artificial greenhouse at 25°C, with a red-blue-yellow LED above the triangular bottle providing photosynthetic photon flux density of 225 μmol m^−2^ s^−1^ and aeration of 1.25 L min^−1^. Under different experimental conditions, the initial concentration of *Chlorella* sp. was 0.26 g L^−1^ (dry weight).

The absorbance of Digestate_M_ at 550 nm was 2.37 ± 0.00 and based on previous results (Koutra et al., 2021), digestate was initially used at 10% (v/v) to research the growth of *Chlorella* sp. without adding nutrient source. In consideration of the large amount of fresh water used in dilution, to reduce the consumption of water resources, part of fresh water was replaced with BWW. In the meantime, the macronutrients and micronutrients in the digestive solution were supplemented in the process. Similar to the pretreatment method of Digestate_M_, after centrifugation, the BWW was first filtered through a 100-mesh filter bag and then filtered again through a sand core filter combined with a 0.45 µm filter membrane. Finally, the physicochemical properties (Table 1) were analyzed, and the BWW was placed at −18°C for subsequent use. The proportion of BWW added was fixed at 20% (v/v), based on Koutra et al. (2019) experimental study on the addition of nutrient elements in similar digestate. When BWW was used to replace part of the deionized water, the proportion of Digestate_M_ still started from 10% and then increased to 30%, 50%, and 80%, during which the proportion of BWW was maintained at 20%. Experimental groups designed according to different dilution ratios of Digestate_M_ included 10% Digestate_M_, 10% Digestate_M_ + 20% BWW, 30% Digestate_M_ + 20% BWW, 50% Digestate_M_ + 20% BWW and 80% Digestate_M_ + 20% BWW, which were all photoheterotrophic cultivation. As a control group, *Chlorella* sp. was also cultivated in 20% BWW in the absence of Digestate_M_. Heterotrophic cultivation in 30% Digestate_M_ + 20% BWW was performed. *Chlorella* sp. cultured in BG11 medium served as the control group for other experimental conditions. In order to simplify the expression, “Digestate_M_” in the name of the experimental group will be omitted in subsequent articles and the expression after omission is shown in Table 2.

**TABLE 2:** Biomass production, average growth rate (µ), and maximum growth rate (µ_max_) of *Chlorella* sp. under different experimental conditions. Data are means ± SD (*n* = 2).

	Culture conditions (v/v)	Biomass production^a^ (g L^−1^)	µ^b^ (d^−1^)	µ_max_ (d^−1^)
Digestate_M_	BG11	1.09 ± 0.02	0.15 ± 0.00	0.49 ± 0.09
	10%	1.18 ± 0.01	0.16 ± 0.00	0.43 ± 0.12
	0% + 20% BWW	0.51 ± 0.02	0.10 ± 0.00	0.41 ± 0.02
	10% + 20% BWW	1.36 ± 0.08	0.17 ± 0.00	0.64 ± 0.01
	30% + 20% BWW	0.87 ± 0.04	0.12 ± 0.00	0.54 ± 0.07
	50% + 20% BWW	0.16 ± 0.01	0.04 ± 0.00	0.19 ± 0.07
	80% + 20% BWW	0.015 ± 0.007	n.d.	n.d.
	30% + 20 BWW (Hetero)	n.d.	n.d.	n.d.

^a^ Calculated as the difference between initial and final values.

^b^Calculated as the difference between initial and final values.

BWW, brewery wastewater; Hetero, heterotrophic conditions; BG11, BG-11 Medium for blue green algae; v/v, Digestate_M_ volume/brewery wastewater volume.

### 2.3 Analytical methods

#### 2.3.1 Determination of biomass concentration and specific growth rate

First, the mass of the centrifuge tube was measured and denoted as m_1_. Then, 10 mL of the sample was centrifuged (D3024, DLAB) to remove the supernatant. Rinse with deionized water for three times and then add 5 mL of deionized water for resuscitation. Density gradient centrifugation was used to separate the suspensions. The gradient medium was 5 mL 50% sucrose solution and 5 mL 15% sucrose solution, centrifuged at 8,000 r min^−1^ for 5 min. The bacteria were located in the upper liquid and *Chlorella* sp. precipitates to the bottom of the centrifuge tube, which was collected separately and vacuum freeze-dried (JC-LDGZ-125, Tsingtao Juchuang) for 24 h. The mass of the centrifuge tube was measured again and denoted as m_2_. The dry weight (m) of *Chlorella* sp. was determined by

$$m=m_{2}-m_{1}$$ (1)

The formula for calculating the specific growth rate of microalgae was as follows:

$$\mu =\frac{\ln m_{t}-\ln m_{0}}{t-t_{0}}$$ (2)

where m_*t*_ is the dry weight at time *t*, and m_0_ is the dry weight at time *t*_0_.

The maximum specific growth rate µ_max_ (d^−1^) is related to the exponential growth period during the growth of microalgae. µ_max_ was estimated by the slope of the logarithmic plot of dry weight concentration versus cultivation time.

#### 2.3.2 Determination of pigment content of *Chlorella* sp.

First, 2 mL of samples were collected at the same time every day during the cultivation of microalgae and centrifuged at 10,000 r min^−1^ for 5 min to remove the supernatant. Then, they were washed with deionized water for three times and centrifuged again to remove the supernatant. Next, the pellet was mixed with methanol and cultured at 45°C away from light for 24 h. Lastly, the supernatant was taken after centrifugation to measure the absorbance. The contents of chlorophyll *a* (Ch a), chlorophyll *b* (Ch b), and carotenoids were calculated using the following formulas (Pancha et al., 2015):

$$Chlorophyll\;a:Ch\;a\;\left(\mu g/ml\right)=16.72\;A_{665.2}-9.16\;A_{652.4}$$ (3)

$$Chlorophyll\;b:Ch\;b\;\left(\mu g/ml\right)=34.09\;A_{652.4}\;–\;15.28\;A_{665.2}$$ (4)

$$Carotenoids=\left(1000\;A_{470}\;–\;1.63\;Ch\;a\;–\;104.9\;Ch\;b\right)/221$$ (5)

The absorbances at 470, 652.4, and 665.2 nm were corrected by subtracting the absorbance at 750 nm.

#### 2.3.3 Digestate_M_ and BWW physicochemical analysis

Digestate_M_ and BWW colors were determined as optical density measured at 550 nm (X-5 UV/VIS MWTASH). pH was determined with a pH meter (PHS–3C, INESA). The COD, TN, TP, and NH_4_^+^-N in Digestate_M_ and BWW were measured using an automatic moisture analyzer (YKM-T4, YOKE). The kits were supplied by the same company. Total solids and volatile solids were measured in accordance with standard methods (Association and Washington, 1995).

#### 2.3.4 Biomass composition analysis

After the experiment, the microalgal solution under various experimental conditions was centrifuged, and the supernatant was removed to keep the algal pellet. The algal pellet was washed 3 times with deionized water and then placed in a vacuum freeze-dryer (JC-LDGZ-125, Tsingtao Juchuang) for 24 h. The freeze-dried algal powder was stored in the dryer at 4°C for following use.

The algal powder was resuspended with phosphate buffer. The microalgal resuspension solution was crushed by 400 W ultrasonic wave (XH-300UA Soniwave, Beijing Xianghu) for 30 min (ice bath) and subsequently centrifuged at 10,000 r min^−1^ for 10 min, and the supernatant was taken. The protein content was analyzed using a BCA protein concentration assay kit (Beyotime, China). The carbohydrate content was analyzed using a carbohydrate content assay kit (Boxbio China).

The lipid of *Chlorella* sp. was extracted using the method of Bligh and Dyer (Bligh and Dyer, 1959). A certain amount of algal powder was weighed and fully ground in a mortar. Microalgal lipids were extracted with mixed organic solvents of chloroform–methanol (2:1; v/v). The mixture phase was oscillated at 150 r min^−1^ in a shaker for 30 min and then centrifuged at 4,000 r min^−1^ for 10 min to recover the supernatant (oil-bearing phase). The above extraction steps were repeated for the ground algal powder several times until the algal powder turned white. All lipid-containing phases were mixed in a 50 mL centrifuge tube that had been dried and weighed and denoted as W_1_ (g). They were then placed in a drying oven (WHL-65B, TAI-SITE) to dry to a constant weight at 37°C and denoted as W_2_ (g). The lipid content (LC) of *Chlorella* sp. was calculated in accordance with the following formula:

$$LC\;\left(\%\right)=\left(W_{2}-W_{1}\right)/W\times 100$$ (6)

where W_2_ (g) is the total weight, W_1_ (g) is the tube weight, and W (g) is the sample weight.

#### 2.3.5 Measurement of photosynthetic fluorescence parameters

F_o_, F_m_, F_s_, F_m_’, F’, F_v_/F_m_, and ΦPSII were directly measured with a pulse modulation fluorometer (PHYTO-PAM-II/ED, WALZ). The electron transport rate (ETR) and effective photochemical quantum yield of PSII [Y(II)] were calculated using the following formulas (Roháek and Barták, 1999):

$$\left[ETR\right]\left(µmol\;m^{-2}\;s^{-1}\right)=PFD\times \Phi PSII\times 0.5\times 0.84$$ (7)

$$Y\left(Ⅱ\right)=\left(Fm’-F’\right)/Fm’$$ (8)

ETR represents the effective photosynthetic ETR, and PFD represents the actual luminous flux density. ΦPSII represents the effective quantum yield of PSII photochemical energy conversion. F_v_/F_m_ represents the maximum quantum yield of PSII photochemistry. PSII minimal fluorescence F_o_ and maximum fluorescence F_m_ were measured after dark adaptation. F_s_ represents the steady-state fluorescence yield, and F_m_’ represents the fluorescence yield after light adaptation; all PSII reaction centers are closed, and all nonphotochemical reactions are at the optimal state. F’ represents the real-time fluorescence yield before performing the saturation pulse.

## 3 Results and discussion

### 3.1 Growth of *Chlorella* sp. under different experimental conditions

**FIGURE 1:**
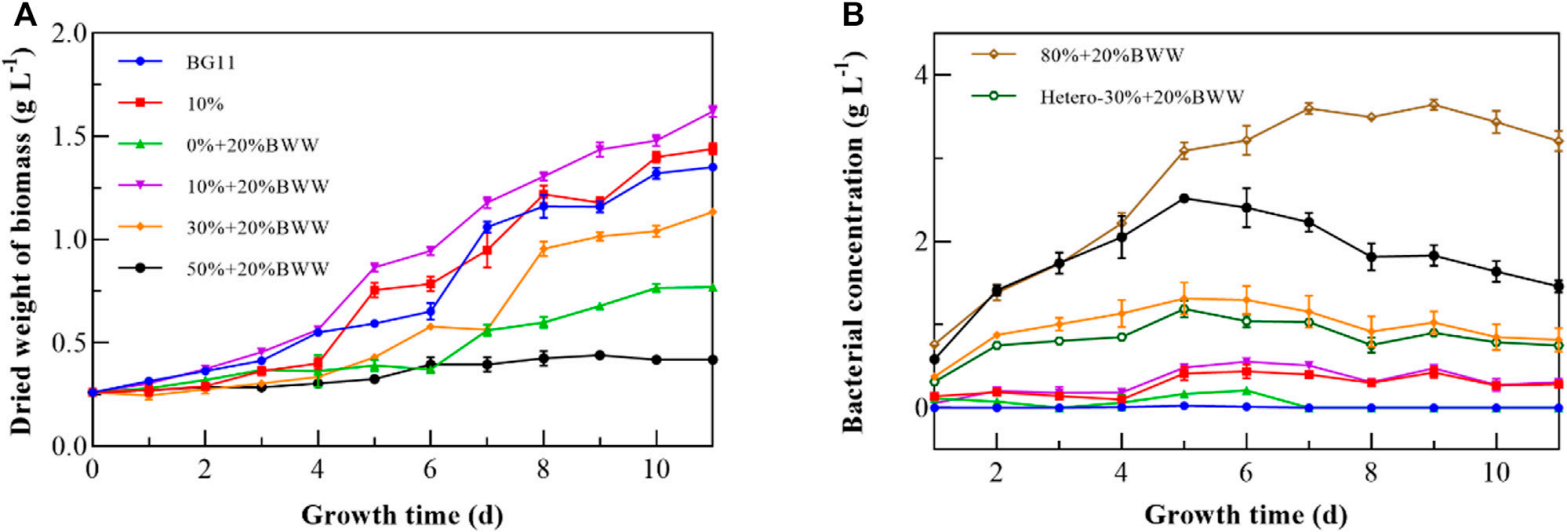
Growth curves of *Chlorella* sp. **(A)** and bacteria **(B)** under different experimental conditions. BWW: brewery wastewater; Hetero: heterotrophic conditions; BG11: BG-11 Medium for blue green algae; 10%: 10% Digestate_M_; 10% + 20% BWW: 10% Digestate_M_ + 20% BWW.

Digestate_M_ from anaerobic fermentation of BWG and BWW was used to cultivate *Chlorella* sp. under different experimental conditions in this study. As shown in Table 1, the COD, pH, TN, and TP of Digestate_M_ were relatively high, and the value of OD_550_ reached 2.37, which indicated a darker color and required a higher dilution ratio before use for *Chlorella* sp. Cultivation (Koutra et al., 2018b). Therefore, the Digestate_M_ ratio started from 10% (v/v); the biomass production of *Chlorella* sp. reached 1.18 g L^−1^ with µ of 0.15 and µ_max_ of 0.43, as presented in Table 2; the incubation time was 11 days under all experimental conditions. When 20% BWW was added, the biomass production and µ_max_ were increased to 1.36 g L^−1^ and 0.64 d^−1^, respectively. As shown in Figure 1A, when only 20% BWW was added, the growth trend was similar to that with 10% + 20% BWW, and a significant reduction in biomass production, by 63.57%, occurred, which was due to the insufficient nutrient of BWW (Chiranjeevi and Mohan, 2016). The BWW addition was maintained at 20%, and the proportion of effluent was increased during the subsequent cultivation process. When the proportion of Digestate_M_ was increased to 50%, the biomass production of *Chlorella* sp. was seriously reduced. When the effluent was increased to 80% loading, the growth of microalge was completely inhibited.

In this study, proliferation of *Chlorella* sp. could not proceed under heterotrophic conditions, which indicated that the present nutrient elements in Digestate_M_ did not allow *Chlorella* sp. to grow without light. Similar to Perez-Garcia et al. (2011) findings, *C. vulgaris* could survive only when organic carbon was added to municipal wastewater under heterotrophic conditions. The reason is that with the increase in the proportion of Digestate_M_, the color darkening reduced the transmittance, which in turn decreased the biomass production (Marcilhac et al., 2014). The inhibitory effect of a high concentration of digestate on microalgae was also reflected in the destructive effect of a high concentration of NH_4_^+^-N on microalgal cells (Collos and Harrison, 2014). However, previous studies have shown that *Chlorella* sp. that was selected in the paper is resistant to high levels of NH_4_^+^-N (Ayre et al., 2017). Bacterial proliferation in digestate, which causes survival competition, is also a factor that inhibits the growth of microalgae and is inevitable (Erkelens et al., 2014), but a certain amount of bacterial growth is acceptable and necessary for microalgae in wastewater (Peccia et al., 2013). The group of 10% + 20% BWW obtained the highest biomass production of 1.40 g L^−1^, which may exert a positive effect by producing symbiotic bacteria that break down large molecules into smaller ones used by microalgae directly (Monlau et al., 2015). Compared with the control group BG11, the biomass production of 10% + 20% BWW was improved by 0.31 g L^−1^, and µ_max_ was increased by 0.15 d^−1^, which might be due to the synergistic effect of symbiotic bacteria. By comparing Figures 1A, B, we can draw the same conclusion that low concentration of symbiotic bacteria corresponds to high growth rate of *Chlorella* sp. From Figure 1B, the endophytic bacteria entered a stable phase from about the fifth day, and the concentration of symbiotic bacteria at this time affected the lag phase of *Chlorella* sp. When the proportion of Digestate_M_ increased from 10% to 30%, the concentration of symbiotic bacteria increased by 2.71 times, which led to the extension of the lag phase. Koutra et al. (2021) found that when the loading of effluent increased to more than 30%, the lag phase increased to about 40 days, accompanied by significant bacterial growth. Accordingly, the growth of *Chlorella* sp. will be inhibited when the concentration of symbiotic bacteria on the fifth day, which is the common starting point of *Chlorella* sp.’s exponential phase and symbiotic bacteria’s stable phase, is about twice that of *Chlorella* sp.

### 3.2 Photosynthetic fluorescence parameters

**FIGURE 2:**
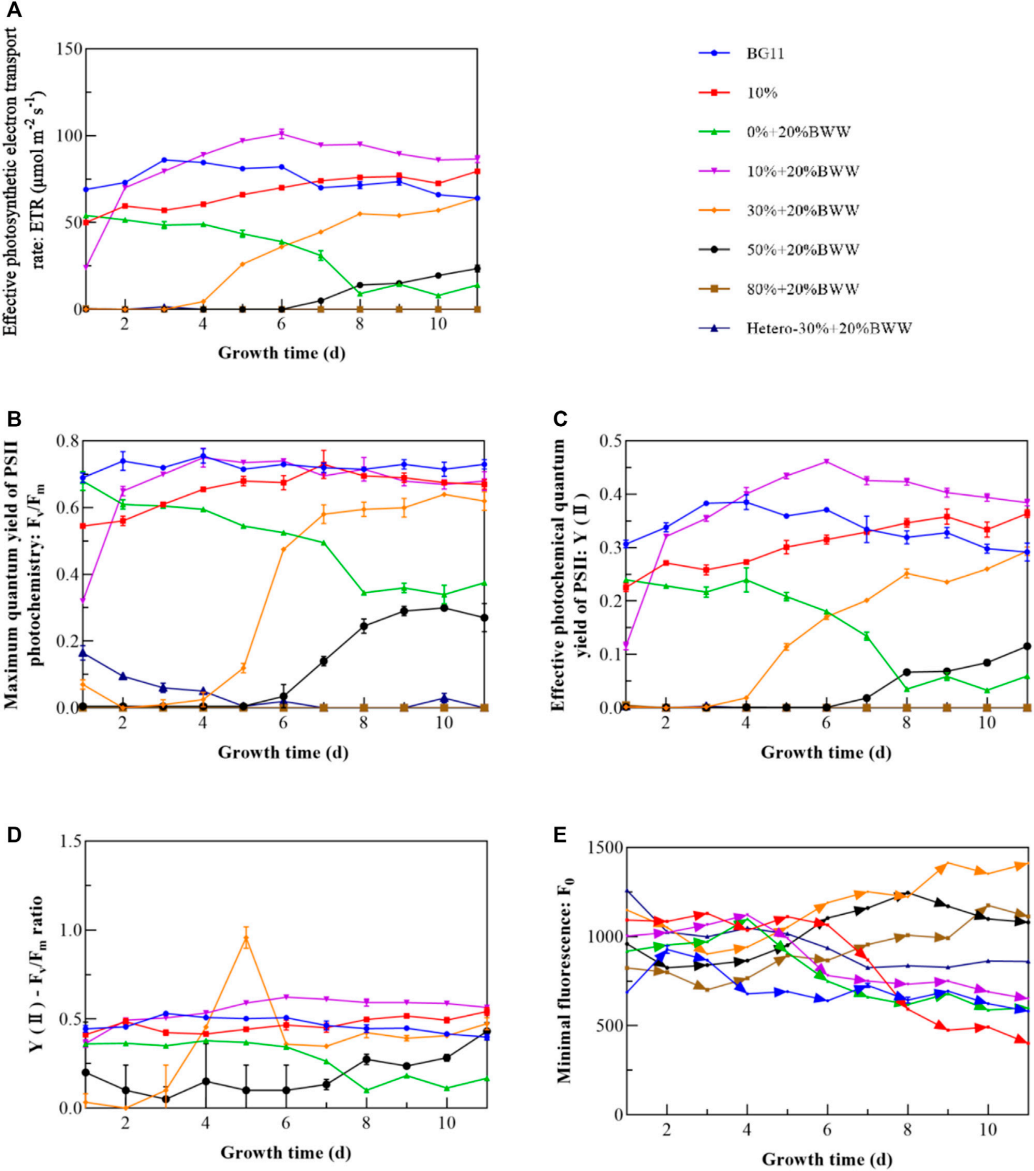
Photosynthetic fluorescence parameters of *Chlorella* sp. under different experimental conditions. **(A)** Effective photosynthetic electron transport rate (ETR); **(B)** maximum quantum yield of PSII photochemistry (F_v_/F_m_); **(C)** effective photochemical quantum yield of PSII [Y(II)]; **(D)** Y(II)–F_v_/F_m_ ratio; **(E)** minimal fluorescence (F_o_). BWW: brewery wastewater; Hetero: heterotrophic conditions. BG11: BG-11 Medium for blue green algae; 10%: 10% Digestate_M_; 10% + 20% BWW: 10% Digestate_M_ + 20% BWW.

Figure 2 shows the variation curves of the photosynthetic fluorescence parameters of *Chlorella* sp. with time under different experimental conditions. Combined with Figure 1, Figure 2 depicts that when *Chlorella* sp.’s growth was not inhibited, ETR (Figure 2A) showed a trend of first increasing and then stabilizing. Group 0% + 20% BWW experienced a rapid reduction in ETR caused by the pressure of nutrient consumption (Hideg et al., 1998). In the early stage of group 20% + 20% BWW, the rapid growth of symbiotic bacteria inhibited the proliferation of *Chlorella* sp., and the inflection point of the rapid increase in ETR was the time node when the symbiotic bacteria entered the stable phase. Subsequently, the rapid increase in ETR greatly improved the biomass yield of *Chlorella* sp. The ETR of 50% + 20% BWW began to increase slowly from zero at the seventh day, which verified that the massive proliferation of bacteria would inhibit the growth of *Chlorella*, resulting in a longer lag phase. F_v_/F_m_ (Figure 2B) and Y (II) (Figure 2C) had similar trends to ETR, which were all positively correlated with microalgal growth curves (Cheng et al., 2019; Xu et al., 2021). Hence, the photosynthetic fluorescence parameters ETR, F_v_/F_m_, and Y (II) can be used to quickly evaluate the final relative value of microalgal concentration in wastewater (Wang et al., 2022). Rapid evaluation of the relative values of microalgal concentration under different experimental conditions is very important for selecting the best culture conditions and for subsequent large-scale applications (Uysal and Ekinci, 2021). Given that most wastewater contains color and particles, the relative concentration of microalgal biomass is difficult to evaluate by spectrophotometry (Cai et al., 2013).

According to F_0_ (Figure 2E), the F_v_/F_m_ values of groups BG11, 10%, and 10% + 20% BWW remained high in the late growth period, mainly due to the rapid reduction in F_0_ and its maintenance at a small value. This result indicated that microalgae can synthesize enough reaction center and maintain a high photochemical quenching potential of PSII under the condition of adequate nutrition and no exogenous bacterial stress. As the F_0_ of 30% + 20% BWW increased, F_v_/F_m_ also increased, probably because F_m_ increased more than F_0_. The analysis of the ratio of Y(Ⅱ) to F_v_/F_m_ (Figure 2D) demonstrated that the average ratio of the experimental groups 10% and 10% + 20% BWW was larger than that of the control group BG11. Thus, when *Chlorella* sp.’s growth was not inhibited in Digestate_M_, mixotrophic cultivation might improve the effective photochemical quantum yield of PSII. Compared with the control group BG11, the Y(II)–F_v_/F_m_ ratio of 30% + 20% BWW and 50% + 20% BWW exceeded that of the control group after the growth of *Chlorella* sp. recovered, indicating that the utilization rate of visible light by *Chlorella* sp. would be significantly improved in an environment with low light transmission rate. From Figures 1A, 2D, the growth of *Chlorella* sp. may be inhibited when the Y(II)–F_v_/F_m_ ratio is less than 0.4.

### 3.3 Pigment characterization

**FIGURE 3:**
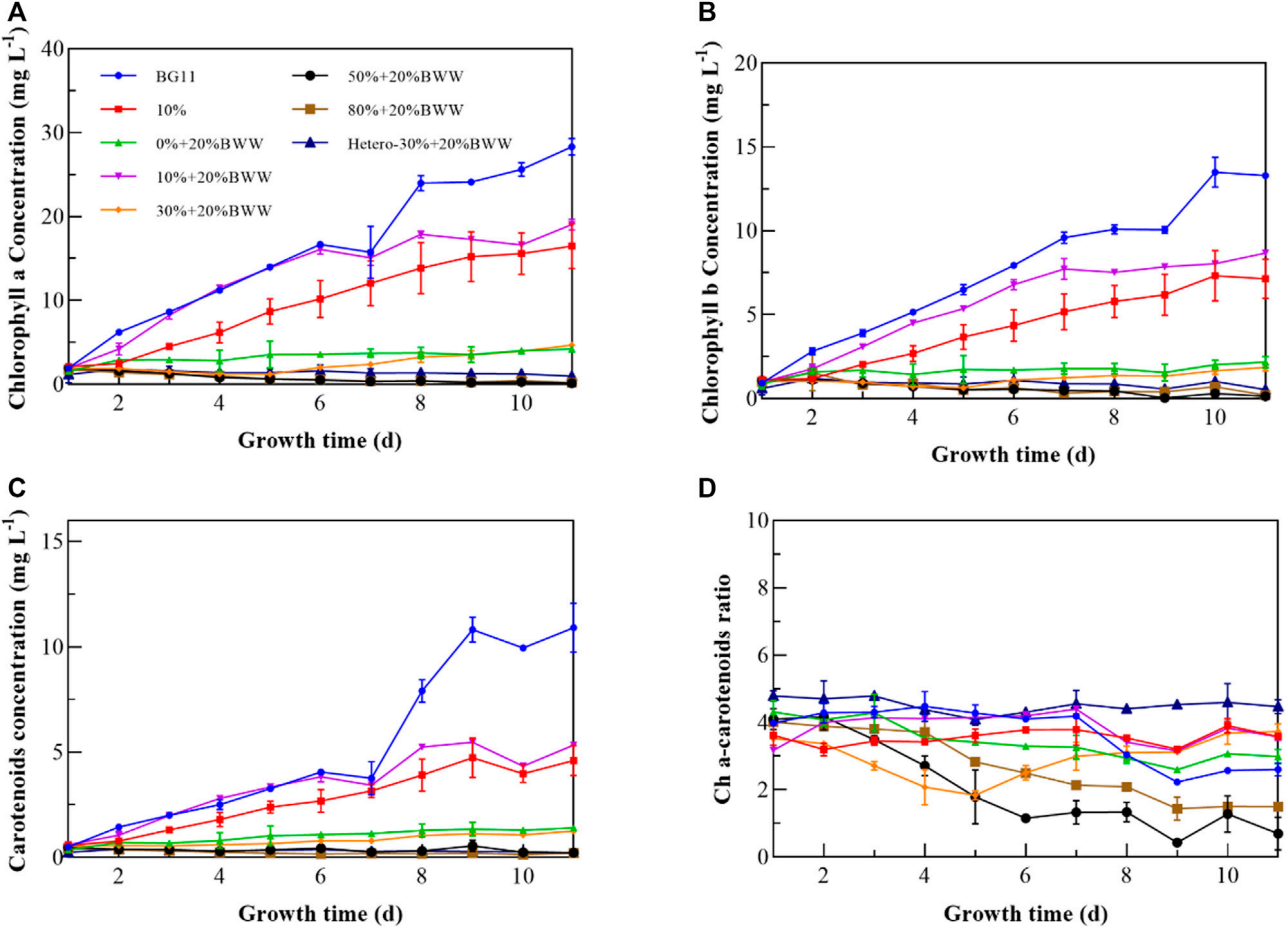
Pigment characterization of *Chlorella* sp. cells under different experimental conditions. **(A)** Chlorophyll a content; **(B)** chlorophyll b content; **(C)** carotenoid content; **(D)** chlorophyll a–carotenoid ratio. BWW, brewery wastewater; Hetero, heterotrophic conditions. BG11, BG-11 Medium for blue green algae; 10%: 10% Digestate_M_; 10% + 20% BWW: 10% Digestate_M_ + 20% BWW.

To study the influence of different culture conditions on the photosynthetic process of *Chlorella* sp., the pigment composition of *Chlorella* sp. was measured throughout the experiments. The change in photosynthetic pigment components is a common stress response of microalgae to environmental change (Park et al., 2015). From Figures 3A, B, excluding heterotrophic cultivation, the chlorophyll content in the experimental group 10% + 20% BWW was the highest (18.49 mg L^−1^), and the lowest was observed for 50% + 20% BWW (0.20 mg L^−1^). When BWW was added to the medium (10% + 20% BWW), the chlorophyll content was increased by 2.75 mg L^−1^ compared with that in the presence of 10% Digestate_M_ alone (10%), which was similar to the previous research result of Yu et al. (2017); that is, the increase in NH_4_^+^-N concentration in the medium was conducive to the accumulation of chlorophyll. The chlorophyll content in BG11 was 27.73 mg L^−1^, the highest among all experimental conditions. Compared with BG11, the experimental group 10% + 20% BWW did not show photoacclimation (Deng et al., 2017), which might be due to the higher ETR and effective photochemical quantum yield of PSII in this experimental group, thus reducing the influence of low irradiance stress. Further analysis showed that when 10% Digestate_M_ was added to the medium (10% + 20% BWW), the chlorophyll content was significantly increased by 16.37 mg L^−1^ compared with that when 20% BWW was added alone, which was similar to the results of Benavente-Valdés et al. (2017). Nevertheless, the results of this paper were contrary to the previous fidings of chlorophyll concentration decrease when organic carbon was added to stimulate heterotrophic metabolism (Ge et al., 2018). The Ch a–carotenoid ratio (Figure 3D) is an important indicator to determine whether microalgae are stressed, and the composition of medium (N stress) has an important relationship with the ratio (Scarponi et al., 2021). In 1988, Hooks et al. (1988) found that the Ch a–carotenoid ratio of microalgae under normal physiological conditions ranged from 2 to 7. In 1994, Becker (1994) reported that nitrogen starvation during the cultivation process would lead to a decrease in chlorophyll and an increase in carotenoids, which provided a theoretical basis for Hooks’s experimental data. Except for group 30% + 20% BWW, the Ch a–carotenoid ratio decreased gradually with the extension of culture time in both autotrophic and mixotrophic cultivation. As shown in Figures 1, 3, the reason why the Ch a–carotenoid ratio of 30% + 20% BWW decreased first and then increased rapidly might be the rapid growth of symbiotic bacteria in the early stage, which then lysed to release nitrogen sources, inducing the endophytic bacteria’s decline period and making *Chlorella* sp. became the dominant group. The Ch a–carotenoid ratio of 50% + 20% BWW and 80% + 20% BWW on the fifth day were 1.78 and 2.83, respectively, indicating severe nitrogen starvation at 50% and 80% effluent ratios, which may provide a way to improve the LC of microalgae (Safi et al., 2014). When the proportion of digestive fluid was 10%, the growth of *Chlorella* sp. was not only normal, but the ratio of Ch a–carotenoid was also higher than that in BG11 (2.60), indicating that the nitrogen source in biogas slurry was relatively sufficient. Photosynthetic pigment was almost not synthesized in heterotrophic culture (Mohammad Mirzaie et al., 2016), which was also the reason why the Ch a–carotenoid ratio of Hetero-30% + 20% BWW was always high.

### 3.4 Nutrient removal from Digestate_M_

**FIGURE 4:**
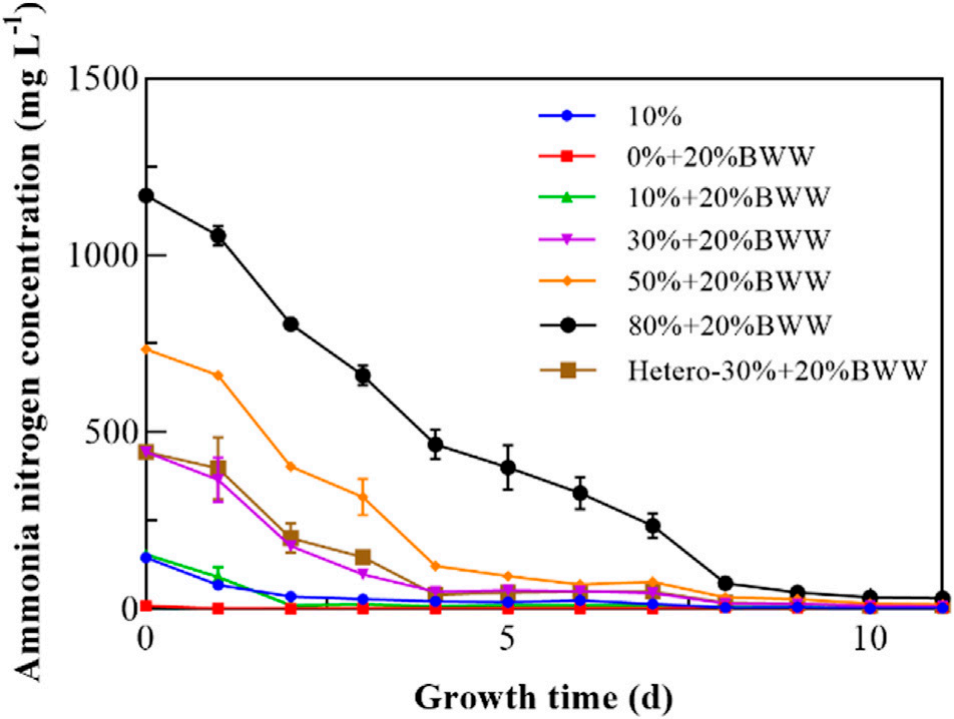
Variation in the concentration of ammonia nitrogen with time. BWW: brewery wastewater; Hetero: heterotrophic conditions. BG11: BG-11 Medium for blue green algae; 10%: 10% Digestate_M_; 10% + 20% BWW: 10% Digestate_M_ + 20% BWW.

In recent years, the effectiveness of microalgal biological process in removing organic matter and nutrients from wastewater is the main target of related research. Ammonia concentration was measured to observe the trend under those test conditions during the microalgal cultivation (Figure 4). As shown in Table 3, except for 0% + 20% BWW, the removal rate of ammonia was higher than 97.00% after 11 days. The highest removal rate of NH_4_^+^-N was 98.20% (30% + 20% BWW), and the maximum removal amount was 1138.8 mg L^−1^ (80% + 20% BWW), which was similar to the previous research results almost achieving complete removal of NH_4_^+^-N. Ledda et al. (2015) treated swine manure obtained by ultrafiltration with wild *Chlorella* sp., and the maximum removal rate for NH_4_^+^-N was 98.00% after 14 days. Franchino et al. (2013) used *C. vulgaris* for nutrition correction of agro-zootechnical digestate, and the NH_4_^+^-N removal rate reached 99.90% after 20 days. Although the removal rate for NH_4_^+^-N in group 0% + 20% BWW was only 68.70%, the remaining amount of NH_4_^+^-N in the medium was only 2.78 mg L^−1^, which was caused by the low initial concentration of ammonia nitrogen. When *C. vulgaris* was used to treat digestate from piggery effluent with an initial concentration of ammonia nitrogen of 20.60 mg L^−1^, the removal rate for NH_4_^+^-N was 54% after 10 days (Jeevan Kumar et al., 2017). Cicci and Bravi (2014) diluted cattle digestate with an initial concentration of ammonia nitrogen of 82 mg L^−1^ by 10 times and cultured *Scenedesmus dimorphus*, but the removal rate for NH_4_^+^-N was only 30%. According to previous experimental results, when the ammonia nitrogen content in wastewater is less than 3–5 mg L^−1^, its concentration is difficult to reduce even if the removal rate is above 99.00% (Massa et al., 2017). To sum up, when the concentration of ammonia nitrogen in wastewater is lower than 5 mg L^−1^, complete removal of ammonia nitrogen can be assumed even if the removal rate is very low. From Figure 4, the ammonia concentration under all experimental conditions dropped rapidly to about 50.00% on the fourth day, which was similar to the experimental result of Scarponi et al. (2021), who found that the ammonia concentration dropped by 50% on the first day because of the air stripping effect. The air stripping effect plays a dominant role in the removal of NH_4_^+^-N under heterotrophic conditions (Ruiz-Martinez et al., 2012; Kim et al., 2016). In this study, the removal rate for ammonia nitrogen under heterotrophic conditions was 97.69%. In the presence of Digestate_M_, the minimum COD removal rate was 87.33% (10% + 20% BWW). With the increase in Digestate_M_ proportion, the COD removal rate gradually reached the maximum, which was 89.89% (80% + 20% BWW). Furthermore, during cultivation in 20% BWW, 121.85 mg L^−1^ of COD was removed (61.22% removal rate), while 10% Digestate_M_ addition resulted in COD consumption of 4.2 g L^−1^, in contrast to 4.0 g L^−1^ in 10% Digestate_M_ alone. When the proportion of effluent was 30%, the COD removal rate of mixotrophic cultivation was only increased by 1.30% compared with that in heterotrophic cultivation, which might be caused by the proliferation of symbiotic bacteria (Praveen et al., 2018) that consumed COD in digestate in the absence of *Chlorella* sp. growth.

**TABLE 3:** Remediation of Digestate_M_ during cultivation of *Chlorella* sp., expressed as NH_4_^+^-N, COD, TN, and TP removal. BWW: brewery wastewater; Hetero: heterotrophic conditions.

Experiment condition	NH_4_^+^-N		COD		TN		TP	
	%_Removal_	mg L^−1^_Removal_	%_Removal_	mg L^−1^_Removal_	%_Removal_	mg L^−1^_Removal_	%_Removal_	mg L^−1^_Removal_
10%	97.59 ± 0.49	141.52 ± 0.71	87.77 ± 0.93	4013.0 ± 42.7	82.22 ± 3.69	168.55 ± 7.57	59.18 ± 1.30	9.64 ± 0.21
0% + 20% BWW	68.70 ± 2.79	6.09 ± 0.25	61.22 ± 0.99	121.85 ± 1.98	62.28 ± 0.56	7.92 ± 0.34	52.38 ± 6.12	0.12 ± 0.01
10% + 20% BWW	97.73 ± 0.92	150.38 ± 1.41	87.33 ± 0.45	4166.7 ± 21.4	86.98 ± 2.96	189.37 ± 6.43	71.86 ± 7.24	11.87 ± 1.20
30% + 20% BWW	98.20 ± 0.48	435.92 ± 2.12	89.04 ± 1.22	12390 ± 170	83.89 ± 2.53	526.57 ± 15.9	65.18 ± 10.08	32.01 ± 4.95
50% + 20% BWW	98.19 ± 0.24	720.70 ± 1.77	89.85 ± 0.93	20718 ± 214	84.22 ± 1.55	873.92 ± 16.1	46.33 ± 1.13	37.85 ± 0.92
80% + 20% BWW	97.41 ± 0.15	1138.8 ± 1.77	89.98 ± 0.99	33091 ± 363	85.05 ± 1.74	1405.6 ± 28.7	42.68 ± 0.70	55.73 ± 0.92
30% + 20 BWW (Hetero)	97.69 ± 0.08	433.67 ± 0.35	87.74 ± 0.61	12209 ± 85.6	78.61 ± 1.14	493.47 ± 7.14	43.09 ± 5.62	21.16 ± 2.76

BG11: BG-11 Medium for blue green algae; 10%: 10% Digestate_M_; 10% + 20% BWW: 10% Digestate_M_ + 20% BWW.

Nitrogen is an essential element in microalgal growth environment. In this paper, the removal rate for TN during cultivation of *Chlorella* sp. varied between 62.28% and 86.98%, observed in 0% + 20% BWW and in 10% + 20% BWW, respectively, and the maximum TN removal concentration was 1.41 g L^−1^ (80% + 20% BWW). However, when the proportion of Digestate_M_ increased to more than 30% and the growth of *Chlorella* sp. was severely inhibited, the TN removal rate could still reach more than 84.00%, which might be caused by the removal of NH_4_^+^-N through the air stripping effect and the growth of heterotrophic microorganisms consuming nitrogen sources (Ledda et al., 2015). Bacteria can remove nitrogen source from digestates and is the main force in removing nitrogen source. By comparing 30% + 20 BWW (Hetero) and 30% + 20% BWW, it can be found that the removal rate of nitrogen source by microalgae is only 5.28%, while the removal rate of endophytic bacteria is 78.61%. Comparison of Tables 2, 3 showed that the removal rate for TP under photoheterotrophic condition was positively correlated with the concentration of microalgal biomass. The TP removal rate of group 10% + 20% BWW with the highest concentration of microalgal biomass was 71.86%; meanwhile, that of group 80% + 20% BWW with severely inhibited growth of microalgae was only 42.68%, which was similar to the result of a previous experiment (Liang et al., 2010). Under the heterotrophic condition, the TP removal rate was 43.09, which was 22.09% lower than that of the control group (30% + 20% BWW), indicating that symbiotic heterotrophic bacteria played an auxiliary role in the absorption of TP (De-Bashan et al., 2002). In conclusion, in the symbiotic system of algae–bacteria, the absorption of TP by microalgae is dominant (de-Bashan et al., 2004; Cruz et al., 2013).

### 3.5 Biochemical composition of *Chlorella* sp. under different regimes

**FIGURE 5:**
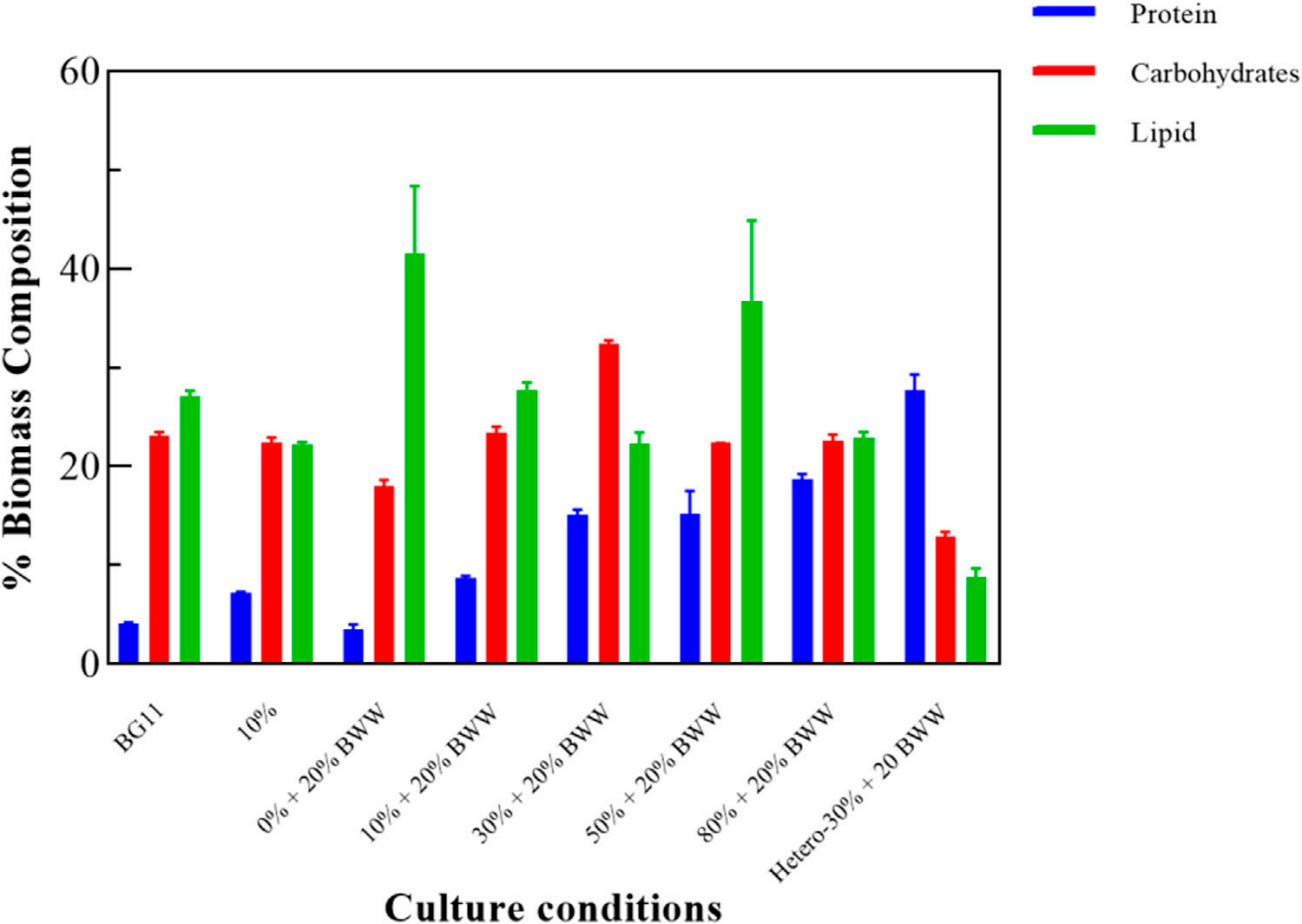
Biochemical composition of *Chlorella* sp. biomass under different experimental conditions. BWW: brewery wastewater; Hetero: heterotrophic conditions. BG11: BG-11 Medium for blue green algae; 10%: 10% Digestate_M_; 10% + 20% BWW: 10% Digestate_M_ + 20% BWW.

*Chlorella* sp. was collected after culture, and its biomass chemical composition was tested to determine its subsequent applications, such as production of biofuels, feed additives, and antioxidant agents. From Figure 5, the main components of *Chlorella* sp. biomass under all photoheterotrophic experimental conditions were carbohydrates and lipids. LC ranged from 19.90% (Herero - 30% + 20% BWW) to 41.60% (0% + 20% BWW) under all experimental conditions, and the LC of the experimental groups 0% + 20% BWW, 10% + 20% BWW (27.80%), and 50% + 20% BWW (36.80%) was higher than that of control group BG11 (27.10%). The highest LC in this experiment was 41.60%, which was 1.60% higher than the 40.00% LC obtained by Yang et al. using domesticated *C. ellipsoideaYJ*1 cultivated in domestic secondary effluent (Jia et al., 2011). This phenomenon might be caused by the absence of nitrogen in the medium (Zhu et al., 2015), and LC increased by 9.00% when the nitrate concentration was reduced from 1.50 g L^−1^ to 0.38 g L^−1^ (Converti et al., 2009). Mujtaba et al. (2012) found that the LC of *Chlorella* sp. was as high as 53% when the concentration of nitrogen source was only 5 mg L^−1^. For further comparison, Li et al. (2011) cultured *Chlorella* sp. in municipal wastewater and obtained 11.00% LC, Qin et al. (2014) cultured *C. vulgaris* in dairy wastewater and obtained 10.30% LC, and Cho et al. (2013) cultured *Chlorella* sp. *227* in wastewater from an anaerobic digestion tank and obtained 10.80% LC, which was about 50% of the lowest LC in photoheterotrophic experimental conditions. Under heterotrophic conditions, the LC was 8.82%, which was slightly higher than Ge et al.'s 5.60% (Ge et al., 2018).

From the analysis of carbohydrates, the carbohydrate content of group 30% + 20% BWW was the highest, which was 9.32% higher than that of the control group BG11 (23.12%). Figure 3D illustrates that the nutrient change curve (the Ch a–carotenoid ratio curve) of group 30% + 20% BWW was similar to that of the fed-batch fermentation method, first decreasing and then increasing, which was conducive to carbohydrate accumulation under the mixotrophic condition (Sivakaminathan, 2012). Meanwhile, the carbohydrate content of group 10% + 20% BWW was slightly higher than that of the control group, and the rest were lower than that, which might be due to the absence of a nitrogen-deficient environment or inhibited growth (Abreu et al., 2012; Goncalves et al., 2018). Nonetheless, all the experimental groups, except group 0% + 20% BWW (3.46%), had a higher protein content than the control group (4.14%), and the highest protein content was 27.72% in group Herero—30% + 20% BWW. Previous experimental results were similar to those in this experiment: The protein content of microalgae under heterotrophic condition was as high as 63.00%, which was 13.20% higher than that in photoheterotrophic condition (Ge et al., 2018). The protein content increased with the increase in digested effluent concentration because a high-nitrogen medium was beneficial to the protein synthesis of microalgae (Zhang et al., 2018).

## 4 Conclusion

Digestate_M_ from anaerobic fermentation of BWG and BWW was effectively used for cultivation of *Chlorella* sp. The concentration of endophytic bacteria was more than twice that of *Chlorella* sp. when they entered the stable stage, which would prolong the lag period of *Chlorella* sp. Further analysis of photosynthetic fluorescence parameters and pigment content showed that photoacclimation did not form with the increase in Digestate_M_ proportion, which might be due to the higher ETR and effective photochemical quantum yield of PSII, thus reducing the influence of low irradiance stress. The growth of *Chlorella* sp. may be inhibited when the Y(II)–F_v_/F_m_ ratio is less than 0.4. The photosynthetic fluorescence parameters ETR, F_v_/F_m_, and Y (II) can be used to quickly evaluate the final relative value of microalgal concentration in wastewater. Based on the results of this study and previous research, when the concentration of NH_4_^+^-N in wastewater is lower than 5 mg L^−1^, complete removal of NH_4_^+^-N can be assumed even if the removal rate is very low. In the symbiotic system of algae–bacteria, the absorption of TP by microalgae is dominant. The protein content increased with the increase in Digestate_M_ concentration.

## Data Availability

The original contributions presented in the study are included in the article/Supplementary Material, further inquiries can be directed to the corresponding author.
